# Experimental data for computing semantic similarity between concepts using multiple inheritances in Wikipedia category graph

**DOI:** 10.1016/j.dib.2020.105377

**Published:** 2020-03-10

**Authors:** Muhammad Jawad Hussain, Shahbaz Hassan Wasti, Guangjian Huang, Yuncheng Jiang

**Affiliations:** a School of Computer Science, South China Normal University, Guangzhou 510631, China; b Division of Science and Technology, University of Education, Lahore, Pakistan

**Keywords:** Semantic similarity, Wikipedia category graph, Multiple inheritances, Information content

## Abstract

This data article compiles the detailed and descriptive experimental data of Wikipedia-based semantic similarity approach called as Neighbourhood Aggregated Semantic Contribution (NASC), presented in Husain, et al. [1]. The JWPL (Java Wikipedia Library)-DataMachine and JWPL WikipediaAPI are used to extract the required Wikipedia features from Wikipedia dump. The dataset presents the disambiguated Wikipedia concepts of the gold standard word similarity benchmarks MC30 (English), RG65_es_ (Spanish) and RG65_fr_ (French) and their associated set of categories in the corresponding Wikipedia category graph (WCG). The dataset also contains the number of ancestors, common ancestors, pages, and common pages in the k-neighbourhood of the associated categories for different levels of parameter *k* in the English, Spanish, and French WCGs. The presented dataset can be used to assess the semantic similarity between Wikipedia concepts in English (MC30), Spanish (RG65_es_), and French (RG65_fr_) languages benchmarks. Moreover, the dataset will be useful for the further analysis and comparison of the taxonomic structures of the English, Spanish, and French WCGs.

**Table:** **Specification Table**

Subject	Information systems
Specific subject area	Artificial Intelligence, Natural Language Processing, Information Retrieval
Type of data	Tables, Graphs, Figures
How data were acquired	JWPL (Java Wikipedia Library)-DataMachine and JWPL WikipediaAPI were used to extract the required information from Wikipedia.
Data format	Raw and processed
Parameters for data collection	We removed cycles and all the hidden categories (administrative categories) from the corresponding Wikipedia category graph.
Description of data collection	We downloaded Wikipedia dump and used JWPL (Java Wikipedia Library)-DataMachine to extract the required features from Wikipedia such as page ids, titles, and page categories. Then we built the corresponding WCG by using JWPL WikipediaAPI. We traversed WCG to get the required data such as: k-neighbourhood, k-ancestors, category pages, and descendant of a particular category.
Data source location	School of Computer Science, South China Normal University, Guangzhou 510631, China.
Data accessibility	Repository name: Mendeley data repository Data identification number: 10.17632/hnmb43sj5s.1 Direct URL to data: http://dx.doi.org/10.17632/hnmb43sj5s.1
Related research article	Author's name: Muhammad Jawad Hussain, Shahbaz Hassan Wasti, Guangjian Huang, Lina Wei, Yuncheng Jiang, Yong Tang Title: “An approach for measuring semantic similarity between Wikipedia concepts using multiple inheritances” Journal: Information Processing & Management DOI: https://doi.org/10.1016/j.ipm.2019.102188

## Value of the Data

• The presented experimental data is useful to measure the semantic similarity between Wikipedia concepts.
• The data is beneficial for all the scientists who are exploiting Wikipedia as a Knowledge Resource.
• The provided data can be manipulated for the further analysis of the taxonomic structures and comparison among the English, Spanish, and French versions of the Wikipedia category graphs.

## 1 Data

**Fig. 1:**
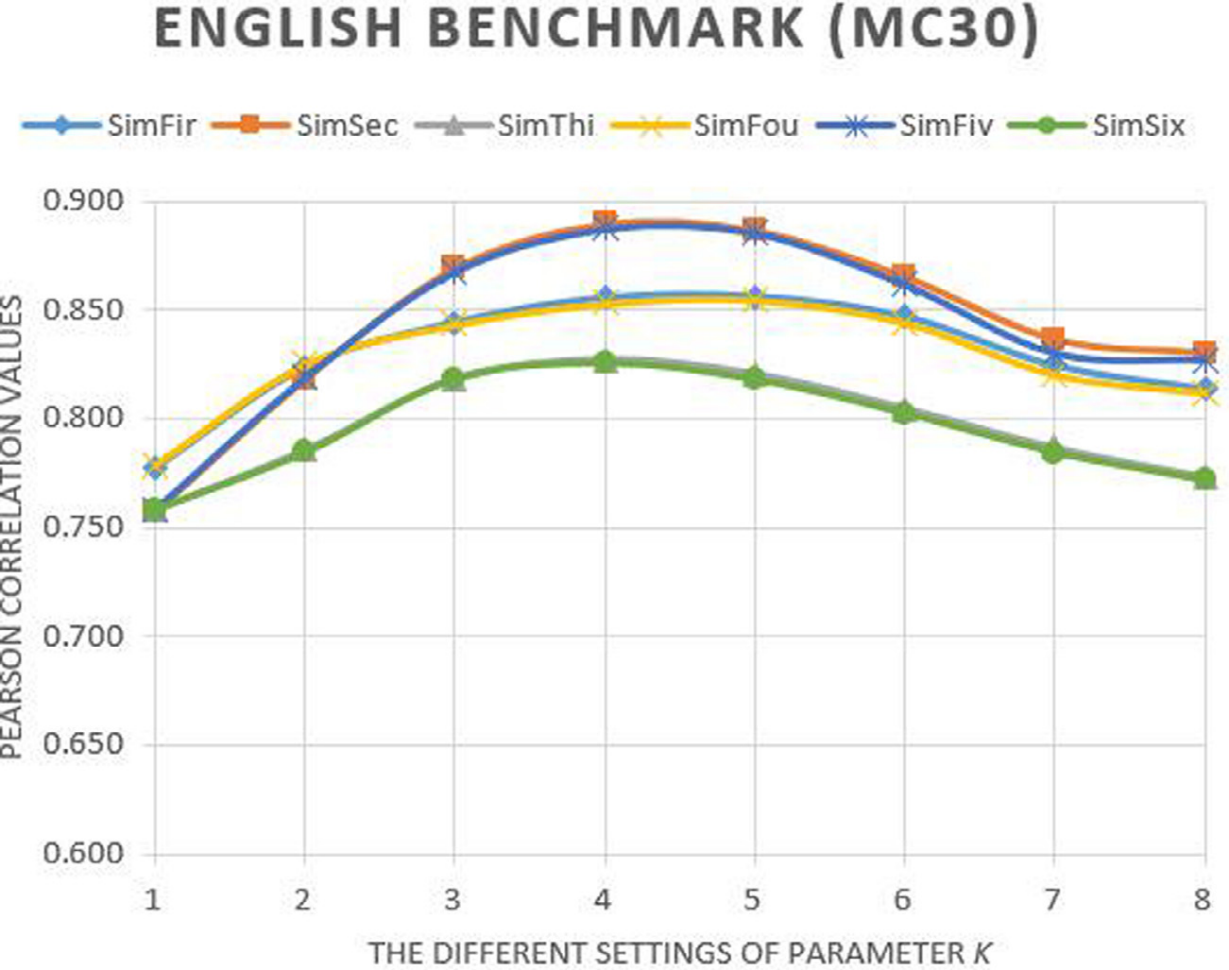
The Pearson correlation of our methods with different settings of parameter *k* on English MC30 benchmark.

**Fig. 2:**
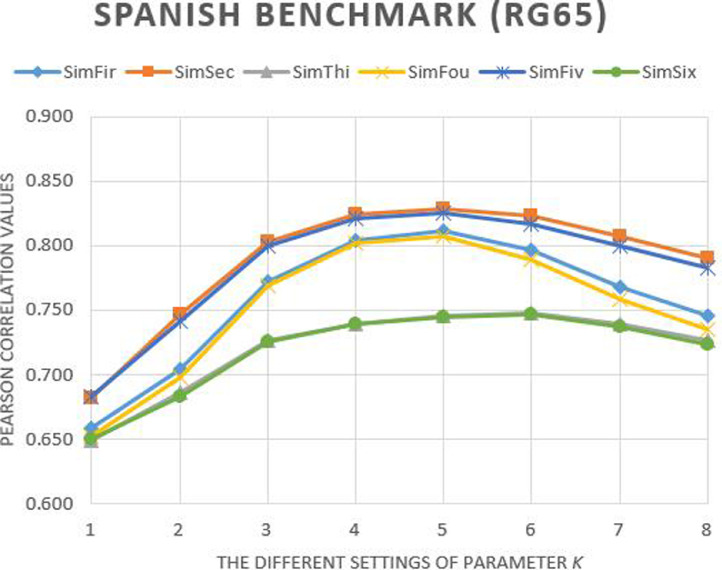
The Pearson correlation of our methods with different settings of parameter *k* on Spanish RG65_es_ benchmark.

**Fig. 3:**
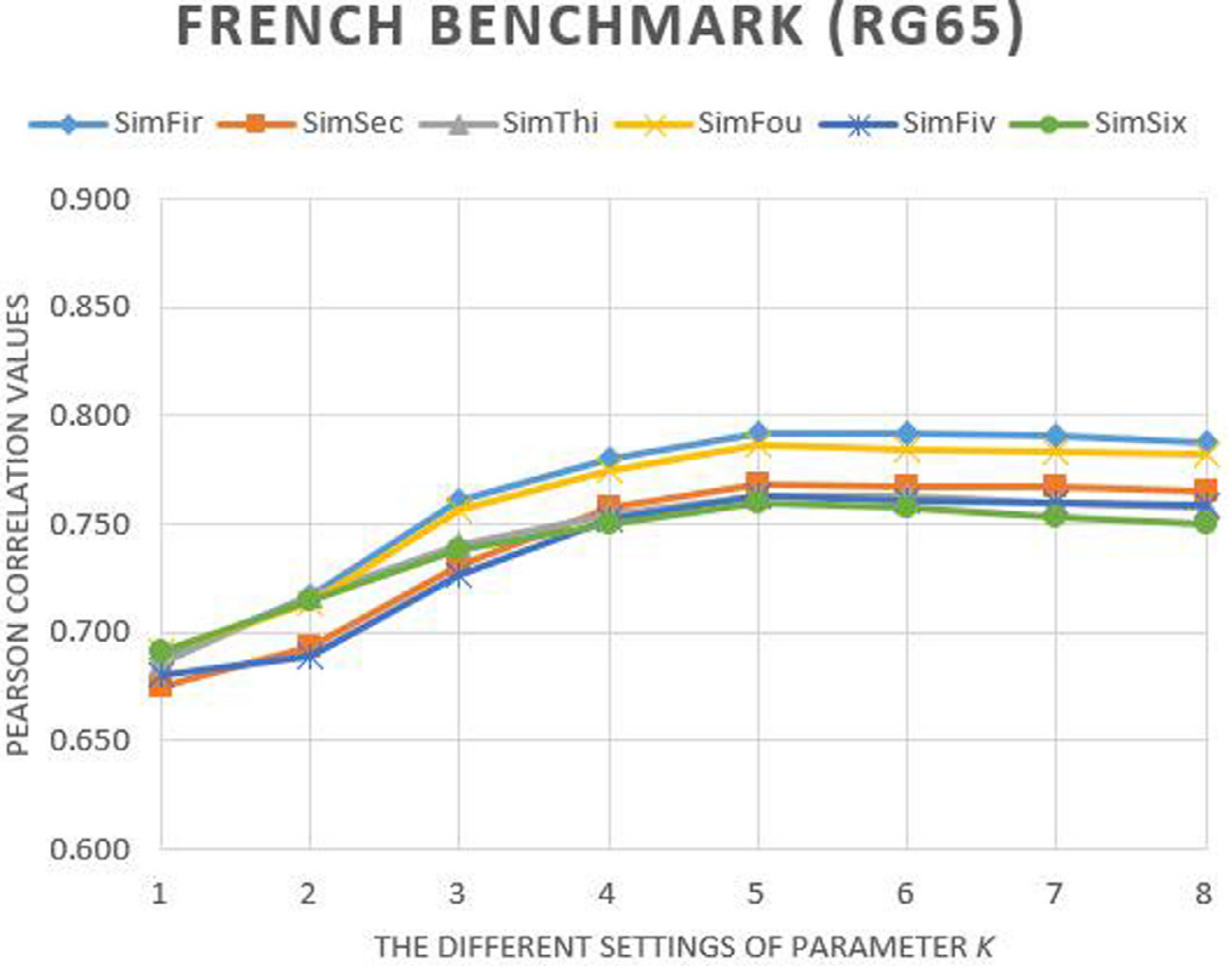
The Pearson correlation of our methods with different settings of parameter *k* on French RG65_fr_ benchmark.

Fig. 1, Fig. 2, Fig. 3 show the graphs of the Pearson correlation values of our proposed Neighbourhood Ancestor Semantic Contribution (NASC)-based semantic similarity methods in gold standard word similarity benchmarks of English, Spanish, and French languages. The Pearson correlation values are shown on different settings of parameter *k* for MC30 (English) [2], RG65_es_ (Spanish) [3], and RG65**_fr_** (French) [4] benchmarks.

Table 1, Table 2, Table 3 present the number of categories and common categories for the selected Wikipedia concept pair (Coast, Forest) from MC30 (English) and its equivalent pairs (Costa, Bosque), and (Cote geographic, Foret) from RG65**_es_** (Spanish) and RG65**_fr_** (French) on different values of parameter *k*. Moreover, these Tables also highlight the structural differences among English, Spanish, and French WCGs in terms of size and branching factor on different values of parameter *k*.

**Table 1:** The number of categories, common categories of English Wikipedia concepts (Coast, Forest) on different settings of parameter *k* using English WCG.

K	Categories of Coast	Categories of Forest	Common categories
1	12	17	0
2	30	54	2
3	62	120	8
4	111	221	22
5	183	356	44
6	267	540	74
7	363	765	124
8	482	999	175

**Table 2:** The number of categories, common categories of Spanish Wikipedia concepts (Costa, Bosque) on different settings of parameter *k* using Spanish WCG.

K	Categories of Costa	Categories of Bosque	Common categories
1	8	9	1
2	18	24	4
3	34	55	12
4	54	101	24
5	78	158	38
6	104	222	51
7	135	286	70
8	173	343	91

**Table 3:** The number of categories, common categories of French Wikipedia concepts (Cote geographic, Foret) on different settings of parameter *k* using French WCG.

K	Categories of Cote geographic	Categories of Foret	Common categories
1	3	18	0
2	5	39	0
3	11	67	2
4	19	101	7
5	31	137	12
6	46	174	21
7	59	206	28
8	69	239	33

**Fig. 4:**
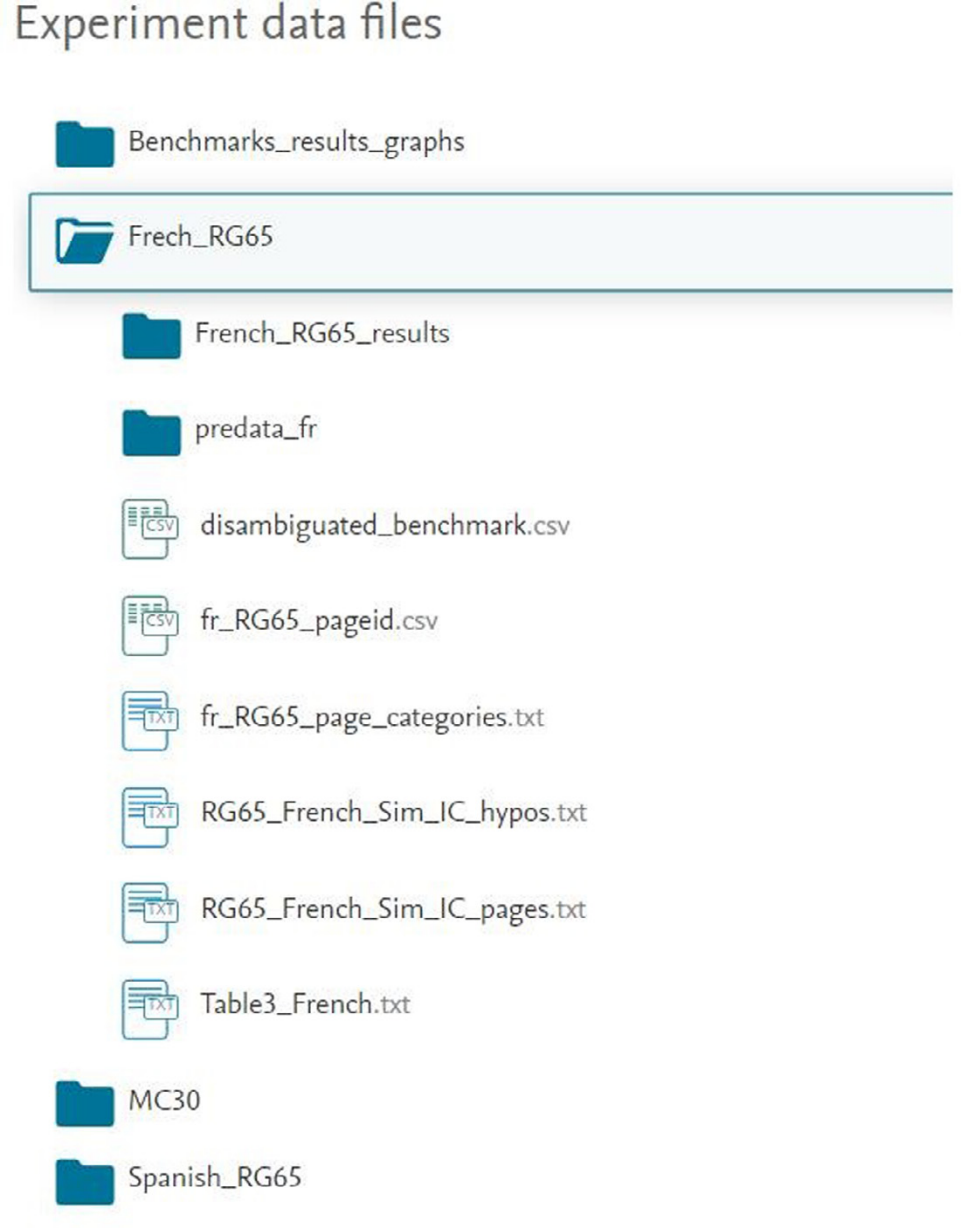
The Mendeley data directory structure of supplementary data files.

Fig. 4 shows the directory structure of all the supplementary data provided with this article on Mendeley data repository [5]. These data files can be used to reproduce the experiments of our methods and for the further analysis on English, Spanish, and French WCGs structures [1]. The folder “Benchmarks_results_graphs” contains all the data related to the graphs that are included in this article. The folders “French_RG65”, “MC30”, and “Spanish_RG65” have all the necessary pre-processed data files to execute the python based program to compute the semantic similarity between English, Spanish, and French Wikipedia concepts according to our methods. For example, as shown in Fig. 4, the folder “French_RG65” contains: (1) the experiments on RG65**_fr_** benchmark in the sub-folder named as “French_RG65_results”, (2) the data required for the computation of $IC_{p}^{k_{neigh}}$ and $IC_{h}^{k_{neigh}}$ [1] in the sub-folder named as “predata_fr”, (3) the disambiguated French Wikipedia concepts in the file named as “disambiguated_benchmark.csv”, (4) the French Wikipedia concepts page ids in the file named as “fr_RG65_pageid.csv”, (5) the French Wikipedia page associated categories in the file named as “fr_RG65_page_categories.txt”, (6) the source code to compute the semantic similarity between the concepts of French Wikipedia using $IC_{h}^{k_{neigh}}$ in the file named as “RG_French_Sim_IC_hypos.txt”, (7) the source code to compute the semantic similarity between the concepts of French Wikipedia using $IC_{p}^{k_{neigh}}$ in the file named as “RG_French_Sim_IC_pages.txt.”, and (8) the source code to reproduce the data associated to Table 3 in the file named as “Table3 _French.txt”.

**Fig. 5:**
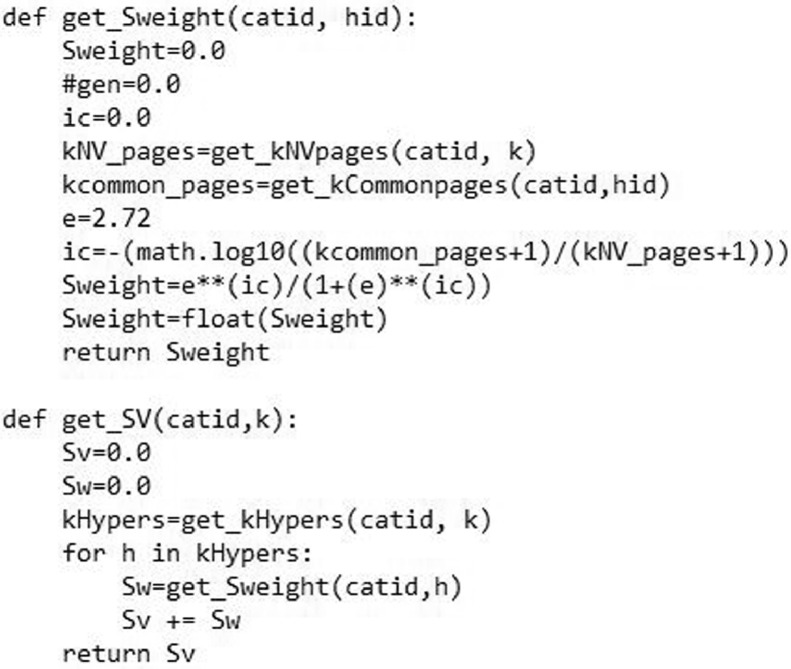
The image of semantic contribution weight and semantic value computation functions.

Fig. 5 shows the image of our python-based functions named as “get_Sweight ()” and “get_SV ()”. These functions are used to compute the semantic weight and semantic value of a category according to its k-neighbourhood in the corresponding WCG respectively.

**Fig. 6:**
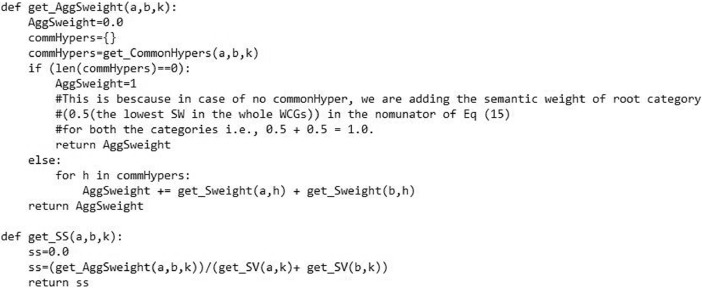
The image of aggregated semantic contribution weight and category similarity computation functions.

Fig. 6 presents the image of our python-based functions named as “get_AggSweight ()” and “get_SS ()”. The first function returns the aggregated semantic weight of a category. The second function computes the similarity between two comparing categories in the corresponding WCG.

**Fig. 7:**
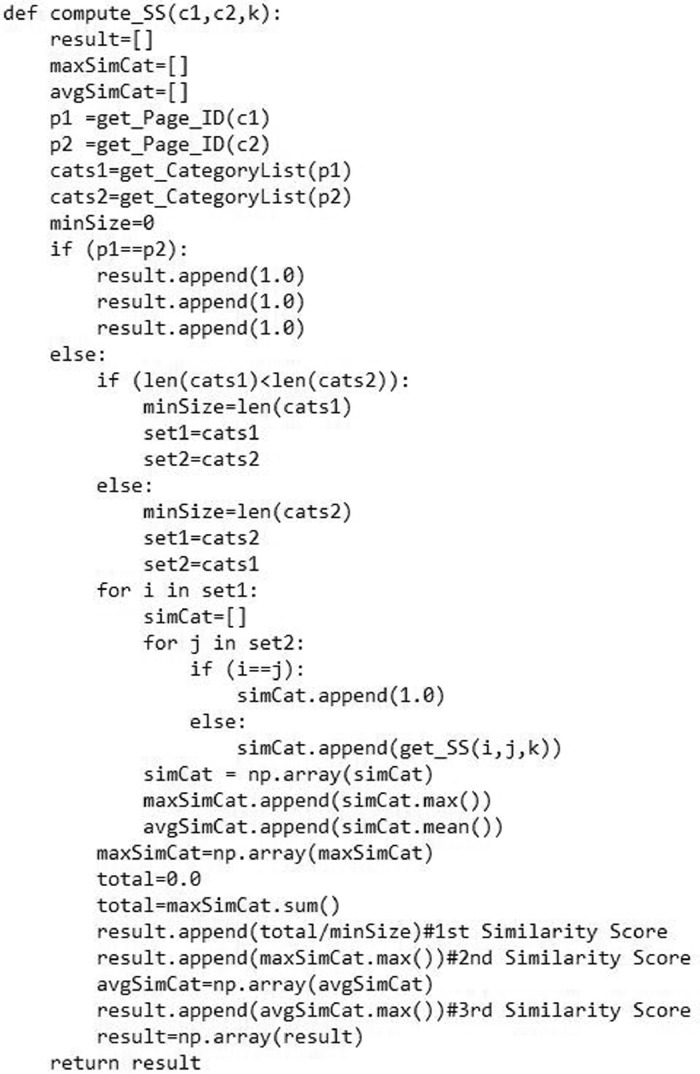
The image of the Wikipedia concepts semantic similarity computation function.

Fig. 7 shows the image of the semantic similarity computation function named as “compute_SS ()”. This function computes the semantic similarity between two Wikipedia concepts for a specific value of parameter *k* in the corresponding WCG.

## 2 Experimental design, materials, and methods

### 2.1 Data extraction

Firstly, we used JWPL (Java Wikipedia Library)-DataMachine to extract Wikipedia features from Wikipedia dump. JWPL is an open-source, Java-based application programming interface that allows access to all the information contained in Wikipedia. JWPL extracts the Wikipedia features such that: page ids, page categories, redirects (synonyms) and category structure etc., from Wikipedia dump file and stores these features in MYSQL tables. Secondly, we constructed Wikipedia category graph (WCG) using JWPL WikipediaAPI. This WikipediaAPI constructs acyclic WCG and removes all the hidden (administrative) categories from it [6]. Finally, we explored the taxonomic structure of this constructed WCG to get the related data such as k-neighbourhood, hypernyms, hyponyms, and k-ancestors. We stored all the required data in the panda data frames to implement our python-based program to compute semantic similarity between Wikipedia concepts.

### 2.2 The parameter *k* and implementation of our methods

We used Wikipedia category graph (WCG) as a semantic network in our methods. However, traversing whole WCG is not only computationally expensive but also reduces the accuracy of multiple inheritance-based semantic similarity methods [1]. Therefore, we only traversed a sub-graph of WCG (referred to as k-neighbourhood) for a particular category (including itself) to define its semantic space. The parameter *k* is a positive integer such that1≤k≤max_depth(WCG), which defines the size of the sub-graph or k-neighbourhood of a category in the corresponding WCG. Intuitively, the k-neighbourhood of a category (node) *a* ∈ *WCG* (k-neighbourhood of (a)) represents the set of all nodes (ancestors or descendants) of the category ‘*a’* which can be traversed via at most *k* edges [7].

We only aggregated the IC-based semantic contribution weights of the k-ancestors of a particular category to achieve the notion of multiple inheritances. Where the k-ancestors represents the ancestors of a category in its k-neighbourhood.

(1) $$k-IC_{p}\left(u\right)=-log\left(\frac{\sum_{v\in k-hyponyms(u)\cup |u|}\left|pages\left(v\right)+1\right|}{\sum_{v\in k_{graph}V\left(u\right)}\left|pages\left(v\right)+1\right|}\right)$$

(2) $$k-IC_{h}\left(u\right)=1-\frac{log(k-hyponyms(u)+1)}{log(|k_{graph}V\left(u\right)|)}$$

(3) $$SC_{w}^{k_{neigh}\left(v\right)}\left(u\right)=\frac{e^{IC^{k_{neigh}\left(v\right)}\left(u\right)}}{1+e^{IC^{k_{neigh}\left(v\right)}\left(u\right)}}$$

We used Eq. (3) to compute the semantic contribution weight of a particular category in the corresponding WCG and assigned a numerical value to it. Note that we implemented Eq. (3) by using two types of ICs (see Eqs. (1) and (2)) [8]. Fig. 5 shows the image of the function which implements Eq. (3). The function “get_Sweight (catid, hid)” returns the IC-based (using Eq. (1) to compute the IC) semantic contribution weight of the ancestor of a category. The function “get_SV (catid, k)” aggregates the semantic contribution weights of all the k-ancestors of a category to compute its semantic value.

(4) $$Sim_{cat}^{k_{neigh}}\left(u,v\right)=\frac{\sum_{w\in (\{k-A(u)\}\cap \{k-A(v)\})}\left(SC_{w}^{k_{neigh(u)}}\left(w\right)+SC_{w}^{k_{neigh(v)}}\left(w\right)\right)}{Sem_{v}^{k_{neigh}\left(u\right)}\left(u\right)+Sem_{v}^{k_{neigh}\left(v\right)}\left(v\right)}$$

To implement Eq. (4), the function “get_AggSweight (a, b, k)” returns the aggregated semantic contribution weight of the common k-ancestors of two comparing categories ‘a‘ and ‘b’ on a specific value of parameter *k*. The function “get_SS (a, b, k)” computes the similarity between two categories by aggregating the semantic contribution weights of the common ancestors of two categories ‘*a’* and ‘*b’* in the nominator and divides it by the individual semantic values of both the categories in the denominator for a specific value of parameter *k* as depicted in Fig. 6.

(5) $$S_{cat}\left(cs_{1},cs_{2}\right)=\frac{1}{min(m,n)}\max_{cs\in CS\left(cs_{1},cs_{2}\right)}\left(\sum_{\langle Cat_{1i},Cat_{2j}\rangle \in cs}Sim_{cat}^{k_{neigh}}\left(Cat_{1i},Cat_{2j}\right)\right)$$

(6) $$MaxS_{cat}\left(cs_{1},cs_{2}\right)=\max_{{\;}_{Cat_{2j}\in cs_{2}}^{Cat_{1i}\in cs_{1}}}\left(Sim_{cat}^{k_{neigh}}\left(Cat_{1i},Cat_{2j}\right)\right)$$

(7) AvgScat(cs1,cs2)=max(Cat1i∈cs1Cat2j∈cs2(Simcatkneigh(Cat1i,Cat2j)))

Finally, the function “compute_SS (c1, c2, k)'' taking three inputs as parameters: the titles of two Wikipedia concepts and the value of parameter *k*. This function computes semantic similarity between two Wikipedia concepts by using different aggregation functions which are defined in Eqs. (5)–(7) [9,10]. These aggregation functions are implemented by using Numpy arrays as depicted below in Fig. 7. The actual source code and all other required data files are provided in the supplementary data.
